# Neurocognitive sequelae following seronegative anti-NMDA receptor encephalitis with ovarian teratoma in a pediatric patient: A six-year follow-up case report

**DOI:** 10.1016/j.bbih.2026.101362

**Published:** 2026-09-17

**Authors:** Felipe Dalvi-Garcia, Iolanda de Salles Fonseca Carvalho, Julia Valeriano de Almeida, Roozemeria Pereira Costa, Ligia Caldeira da Silva, Julia Nunes Perez Fandiño

**Affiliations:** a Institute of Psychiatry, Federal University of Rio de Janeiro, Venceslau Brás Avenue, 71, Botafogo, Praia Vermelha Campus, Rio de Janeiro, RJ, 22290-140, Brazil; b Servidores do Estado University Hospital, Brazilian Ministry of Health, Sacadura Cabral Street, 178, Saúde, Rio de Janeiro, RJ, 20221-161, Brazil; c Fernandes Figueira Institute, Oswaldo Cruz Foundation, Rui Barbosa Avenue, 716, Flamengo, Rio de Janeiro, RJ, 22250-020, Brazil; d Albert Einstein Israelite Institute for Education and Research, Comendador Elias Jafet Street, 755, Morumbi, São Paulo, SP, 05653-000, Brazil

**Keywords:** Anti-NMDA receptor encephalitis, Autoimmune encephalitis, Seronegative, Ovarian teratoma, Neuroinflammation, Autism spectrum disorder

## Abstract

**Background:**

Anti-N-methyl-D-aspartate receptor (NMDAR) encephalitis is the commonest form of autoimmune encephalitis of childhood. However, little information is captured with formal neuropsychological testing. When the pre-illness history is incomplete, it is also hard to judge how much of the residual picture reflects the encephalitis itself rather than an earlier neurodevelopmental vulnerability.

**Case presentation:**

We describe the case of an adopted girl who became acutely unwell at 11 years of age, with a rapid neuropsychiatric deterioration, seizures and abnormal movements. A mature ovarian teratoma was found and removed, and the clinical course, serial EEGs and response to immunotherapy supported a diagnosis of probable anti-NMDAR encephalitis. Serum and cerebrospinal fluid (CSF) antibodies were negative, although the samples were taken late and after treatment had begun. She was managed with corticosteroids and intravenous immunoglobulin. Six years on, formal testing showed largely intact verbal comprehension but clear weaknesses in executive function, attention, processing speed and verbal memory. She met criteria for autism spectrum disorder; overall cognitive ability was in the low average range.

**Conclusion:**

This case shows how a paraneoplastic, immune-mediated insult to the developing brain can leave a lasting cognitive mark, how easily seronegative disease is missed, and why it is often neuropsychological assessment, rather than imaging, that reveals the residual burden.

## 1 Introduction

Anti-NMDAR encephalitis sits at the interface of neurology and psychiatry. Its acute phase is well characterized in children, but long-term neuropsychological outcomes documented by formal testing remain scarce in the case-report literature, and the distinction between encephalitis sequelae and preexisting neurodevelopmental conditions is particularly difficult to resolve when pre-illness developmental data are incomplete.

Autoimmune encephalitis (AE) is a rare (estimated incidence of 0.8/100,000/year) heterogeneous group of immune-mediated disorders of the central nervous system, of which anti-NMDAR encephalitis is the most prevalent subtype in children (Cellucci et al., 2020; Dubey et al., 2018). Approximately 37% of anti-NMDAR encephalitis cases present before 18 years of age (Titulaer et al., 2013). It typically presents with psychiatric symptoms such as mania, delusions, hallucinations, agitation, and behavioral dyscontrol, which often precede seizures, movement disorders, and autonomic instability by weeks to months (Dalmau et al., 2008; Dalmau and Graus, 2018; Honnorat and Plazat, 2018). The association with ovarian teratomas in adolescent and young adult females is well established, and combined immunotherapy with tumor resection yields superior functional outcomes compared with immunotherapy alone (Liu et al., 2022; Titulaer et al., 2013). Diagnosis remains challenging, since approximately 13% of patients present with seronegative cerebrospinal fluid (CSF) findings, particularly when lumbar puncture is performed at advanced stages or after immunotherapy initiation (Dutra et al., 2018; Kaneko et al., 2018). An underrecognized aspect of pediatric anti-NMDAR encephalitis is the persistent neurocognitive sequelae that emerge despite clinical remission, often including deficits in executive function, attention, working memory, and social cognition (Finke et al., 2012; Titulaer et al., 2013).

The aim of this report is to describe the long-term neuropsychological outcome of pediatric seronegative anti-NMDAR encephalitis and to draw attention to the diagnostic pitfalls of seronegative, teratoma-associated disease. We present an adopted girl in whom the diagnostic trajectory, pharmacological management and structured neuropsychological assessment six years after symptom onset are detailed. Her incomplete pre-illness developmental record offers an unusual opportunity to consider the boundary between encephalitis sequelae and preexisting neurodevelopmental conditions.

## 2 Case report

The patient was an adopted girl of mixed-race (*parda*) background - in the Brazilian official classification, the Black population comprises black (*preta*) and mixed-race (*parda*) people - with limited prenatal and early developmental records; her biological family history reportedly included a mother with schizophrenia and several siblings with unspecified severe psychiatric disorders. In early 2018, she developed an insidious neuropsychiatric syndrome that progressed over several months to include mutism, postural abnormalities, severe behavioral disorganization, and episodes initially interpreted as absences but later characterized as focal seizures with impaired awareness. These changes represented a marked departure from her baseline functioning. Earlier assessments (from around six years of age) had produced several provisional psychiatric diagnoses: attention-deficit/hyperactivity disorder (2015), autism spectrum disorder (ASD) (2018, unconfirmed), childhood-onset bipolar disorder (2019), and early-onset schizophrenia. Sequential trials of stimulants, antipsychotics, and mood stabilizers (including risperidone, haloperidol, sulpiride, carbamazepine, methylphenidate and biperiden, and, from January 2019, sodium valproate and olanzapine; doses before referral were not systematically documented) had yielded inadequate or only partial response. These earlier evaluations were predominantly psychiatric; neuroimaging was not undertaken at that stage, and brain MRI, although later requested, was obtained only in 2024.

During this period, an endocrine workup for early pubertal development (investigated from late 2017) identified a left ovarian mass on pelvic ultrasonography, with imaging features suggesting a dermoid cyst. A left oophorectomy in November 2018 confirmed a mature cystic teratoma. Neuropsychiatric symptoms nonetheless continued to worsen after tumor resection, arguing against mass effect and pointing toward an underlying neuroimmunological process.

In 2019, at 11 years of age, she presented acutely to a child psychiatric service with irritability, psychomotor agitation, aggression toward others, delusions, hallucinations, rapid skill deterioration, and focal seizures. On admission, postural abnormalities and motor coordination deficits were noted, with no other systemic findings. At that admission she was receiving sodium valproate (500 mg/day), aripiprazole (10 mg/day) and olanzapine (15 mg/day). Given this progression, now reinforced by the recently resected teratoma, the neuropediatric service considered AE.

The presentation met both pediatric criteria for probable antibody-negative AE (Cellucci et al., 2020) and adult-validated criteria for probable anti-NMDAR encephalitis (Cellucci et al., 2020; Graus et al., 2016); the pediatric criteria of Cellucci et al. (2020) provide the child-specific framework applied here, and both criterion sets are summarized in Table 1. CSF analysis showed mild pleocytosis (10 cells/mm^3^) with a negative IgG index; pleocytosis constituting the required paraclinical evidence of neuroinflammation under the pediatric criteria and a supportive finding under the adult criteria. Antibody testing was undertaken in both serum and CSF at a reference neuroimmunology laboratory (Medical University of Vienna): screening by tissue-based assay (avidin-biotin-peroxidase technique, rat brain) and indirect immunofluorescence (IIFT, Euroimmun) showed no neuropil staining pattern typical of neuronal surface antibodies (including NMDAR, LGI1, CASPR2, AMPAR, GABA-B receptor and DPPX), and an immunoblot for onconeuronal antibodies and cell-based assays for glycine-receptor and MOG antibodies were likewise negative, though seronegativity was not unexpected given that testing was performed at an advanced stage (more than 18 months after symptom onset) and after immunotherapy initiation. Brain MRI was requested but not obtained during the acute workup; a delayed MRI performed in 2024 showed no structural abnormalities. Serial EEGs demonstrated extensive background slowing with subcortical dysfunction and bilateral frontopolar epileptiform abnormalities, with progressive improvement on follow-up recordings.

**Table 1:** Summary of the diagnostic criteria applied in this case. In the pediatric criteria, EEG abnormalities may substitute for the clinical feature of altered mental status but do not count as paraclinical evidence of neuroinflammation. NMDAR, N-methyl-D-aspartate receptor; AE, autoimmune encephalitis; CSF, cerebrospinal fluid; CNS, central nervous system; EEG, electroencephalogram.

Probable antibody-negative pediatric autoimmune encephalitis (Cellucci et al., 2020)	Probable anti-NMDAR encephalitis (Graus et al., 2016)
Acute or subacute (< 3 months) onset in a previously healthy child, with clinical evidence of neurologic dysfunction - at least 2 of: • altered mental status/level of consciousness (or EEG showing slowing or epileptiform activity); • focal neurologic deficits; • cognitive difficulties; • acute developmental regression; • movement disorder (except tics); • psychiatric symptoms; • seizures not explained by a previously known seizure disorder or other condition.	Rapid onset (< 3 months) of ≥ 4 of six symptom groups: • abnormal behavior/cognition; • speech dysfunction; • seizures; • movement disorder/dyskinesia/rigidity; • decreased consciousness; • autonomic dysfunction or central hypoventilation.
Paraclinical evidence of neuroinflammation - at least 1 of: • CSF inflammatory changes (leukocytosis > 5 cells/mm^3^ and/or oligoclonal bands); • MRI features of encephalitis; • brain biopsy showing inflammatory infiltrates and excluding other disorders.	Paraclinical evidence of neuroinflammation - at least 1 of: • abnormal EEG (focal/diffuse slow or disorganized activity, epileptic activity, or extreme delta brush); • CSF pleocytosis or oligoclonal bands.
No well-characterized autoantibodies associated with AE in serum or CSF, and reasonable exclusion of alternative causes, including other causes of CNS inflammation.	Reasonable exclusion of alternative causes. (Diagnosis may also be made with three symptom groups plus a systemic teratoma.)

Treatment began with high-dose intravenous methylprednisolone (30 mg/kg) followed by an oral prednisone taper (2 mg/kg/day) from August 2019 to February 2020, with partial improvement. Intravenous immunoglobulin (IVIG; 2 g/kg total) was then administered as five doses at two-week intervals followed by two monthly doses, with subsequent stabilization of seizures and behavior. Valproate (500-750 mg/day) was transitioned to topiramate (400 mg/day, tapered progressively and withdrawn in 2023) for seizure control. Persistent psychosis was managed with clozapine (titrated to 300 mg/day and reduced to 100 mg/day by 2024, the most recently documented dose), which proved effective without hematologic complications. Nortriptyline (10 mg/day) was added for chronic headaches and methylphenidate (10 mg/day) for persistent attentional dysfunction. The dosages and regimens of all medications used over the course of follow-up are illustrated in Fig. 1.

**Fig. 1:**
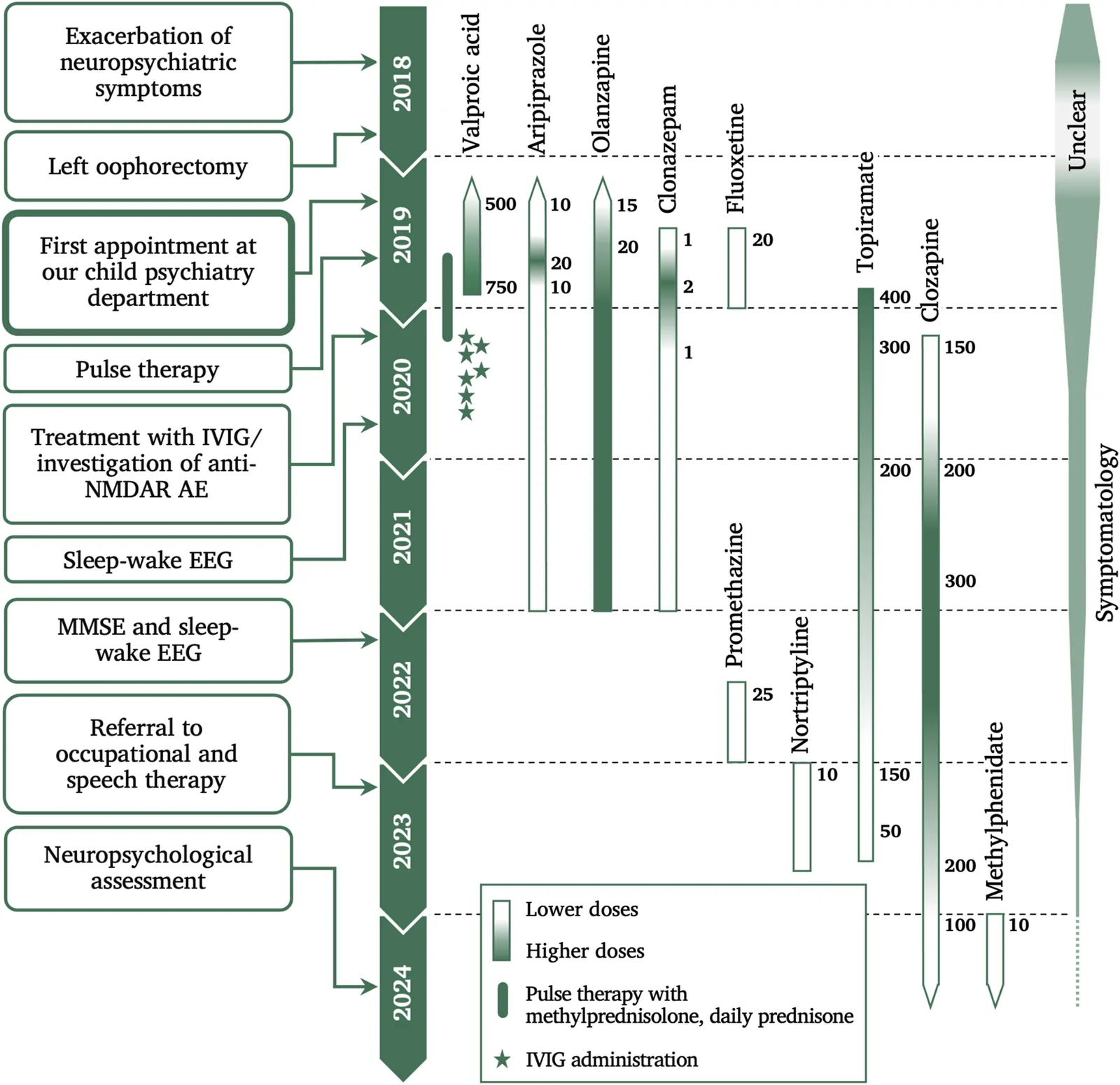
Timeline of key events in the reported case. MMSE: Mini-Mental State Examination. EEG: electroencephalogram. IVIG: intravenous immunoglobulin (2 g/kg). Pulse therapy: high-dose intravenous methylprednisolone (30 mg/kg) followed by a daily oral prednisone (2 mg/kg) taper. Numbers beside the medication bars indicate doses in mg/day. Triangular ends denote treatment initiated before, or continuing beyond, the period depicted.

In the months following IVIG initiation, seizure control and disorganization improved, although residual psychotic phenomena including delusional content and behavioral disinhibition persisted intermittently for over a year. By 2022, follow-up EEG showed measurable improvement in background activity relative to earlier tracings, and behavioral adaptation advanced gradually with structured educational and community-based interventions.

Functional capacity, emotional regulation, and adaptive functioning improved gradually, although significant residual difficulties persisted in social interaction and cognition. The adoptive mother, a teacher, was the principal mediator of care: she worked at the patient's school, accompanied her in the classroom and implemented strategies at home, and was central to history-gathering and to the diagnostic and rehabilitation process. Educational accommodation required intensive school-based support, structured home interventions, individual and family psychotherapy, occupational therapy, and speech and language therapy coordinated through community mental health services (a community child mental-health service, CAPSi), together with an adapted curriculum and classroom mediation. The stated objectives were to strengthen phonological awareness and oral-language development, to build vocabulary and reciprocal social communication, and to establish daily-living routines (for example, structured routine charts for self-care). The 2024 assessment additionally recommended structured neuropsychological and psychopedagogical rehabilitation, targeting attention, inhibitory control, working memory, planning, organization and academic skills, and consideration of an individualized education plan; their subsequent implementation could not be verified from the available records.

Neuropsychological assessment in 2024, including Wechsler Intelligence Scale for Children 4th Edition (WISC-IV) (Wechsler, 2013), Rey-Osterrieth Complex Figure (Oliveira and Rigoni, 2010), Five Digit Test (FDT) (Paula and Malloy-Diniz, 2015), Psychological Battery for Attention Assessment (BPA) (Rueda, 2013), Rey Auditory Verbal Learning Test (RAVLT) (Magalhães and Hamdan, 2010), and School Achievement Test 2nd Edition (TDE-II) (Stein et al., 2019) showed low average overall cognitive ability (Full-Scale IQ 89, 23rd percentile) with marked variability across domains. Verbal comprehension was preserved (index score 106, 66th percentile), while perceptual reasoning (index score 88) and processing speed (index score 86) fell below average and working memory was borderline (index score 77). Executive functions including inhibitory control, cognitive flexibility, sustained attention, and planning were globally impaired. She performed at or below the first percentile on divided and alternating attention tasks. Verbal episodic memory was poor, with failure to recognize post-interference stimuli. Academic abilities were significantly below age and grade expectations, and she showed moderate symptoms of impaired social-communication and restricted behaviors.

This profile matched established sequelae of pediatric AE in executive function, attention, and memory domains. The constellation of cognitive, behavioral, and social-communicative findings prompted formal evaluation for neurodevelopmental disorders. Social-communication and restricted, repetitive behaviors were quantified with the Social Responsiveness Scale, Second Edition (SRS-2) (Constantino, 2012), completed by school staff, and used as a standardized measure of autism-related characteristics integrated with the developmental history, caregiver interview, school information and clinical observation, rather than as an isolated diagnostic instrument; gold-standard diagnostic instruments (ADOS-2, ADI-R) were not administered. On this integrated formulation, criteria were met for ASD (ICD-11 6A02), classified on clinical judgement as requiring substantial support (level 2). Attention-deficit/hyperactivity symptoms were assessed with a parent-rated scale (ETDAH-Pais) (Benczik, 2018), on which hyperactivity/impulsivity fell within the average range (55th percentile) while the attention factor was moderately elevated (70th percentile); objective testing showed concentrated attention at the 10th percentile and divided, alternating and general attention at the 1st percentile. These findings were interpreted as significant attentional and executive alterations within the broader neuropsychological picture; an independent diagnosis of ADHD was not established, although a dedicated exclusion protocol was not undertaken. The assessment also recorded a clinical hypothesis of a mild co-occurring disorder of intellectual development; because no standardized adaptive-functioning measure (for example, Vineland or ABAS) was administered, this could not be confirmed, and the heterogeneous profile (low average Full-Scale IQ with preserved verbal comprehension) counsels caution in that attribution.

## 3 Discussion

This case illustrates three features that make pediatric anti-NMDAR encephalitis particularly difficult to characterize: seronegativity following advanced-stage testing, the impossibility of distinguishing acquired sequelae from preexisting neurodevelopmental vulnerability when pre-illness data are absent, and the persistence of multidomain cognitive impairment documented by structured neuropsychological testing six years after symptom onset. A central unresolved question is whether the current neurodevelopmental diagnosis reflects sequelae of encephalitis-related injury or preexisting conditions not previously documented.

The 2018 presentation, dominated by psychotic symptoms, led to hypotheses of bipolar disorder and childhood-onset schizophrenia (Dalgalarrondo, 2018). Definitive treatment came months later, when fluctuating attention and consciousness with disorientation and confused thinking pointed toward a neurocognitive disorder. This delay mirrors the broader literature showing that pediatric anti-NMDAR encephalitis often begins with psychiatric symptoms, postponing neurological workup (Cellucci et al., 2020; Graus et al., 2016; Titulaer et al., 2013).

The subsequent emergence of mutism, postural abnormalities, and seizures shifted suspicion toward AE, and the ovarian teratoma strengthened the diagnosis via the well-established paraneoplastic association in female patients (Graus et al., 2016; Titulaer et al., 2013). The extended delay mirrors a common pattern in which medical conditions in psychiatric patients remain unrecognized for long periods, with healthcare access barriers contributing to morbidity through missed or late detection (Bradford et al., 2008; Kågström et al., 2025; Liu et al., 2017).

The ovarian teratoma provides a plausible immunological trigger. Teratomas frequently contain neural tissue that expresses NMDA receptors; ectopic expression of GluN1/GluN2 subunits within the tumor is thought to break immune tolerance and drive production of anti-GluN1 antibodies that subsequently target the brain (Dalmau et al., 2008, 2019). This paraneoplastic mechanism is most often recognized in adolescent and young-adult females, in whom an underlying ovarian teratoma is identified in a substantial proportion of cases, and in whom tumor removal combined with immunotherapy yields better functional outcomes than immunotherapy alone (Liu et al., 2022; Titulaer et al., 2013). Ethnicity may also be relevant. Population-based data from the United States indicate a substantially higher incidence of anti-NMDAR encephalitis in Black, Hispanic and Asian/Pacific Island persons than in White persons, with ovarian teratomas identified in more than half of affected Black female patients (Alsalek et al., 2024); disproportionate incidence has likewise been reported in Māori and Pacific Island children (Jones et al., 2017) and in Austronesian populations (Wong et al., 2021). Our patient's background is consistent with these epidemiological patterns, although comparable Brazilian data are lacking and no causal inference can be drawn from a single case.

A notable feature of this case is the negative CSF result despite a presentation highly consistent with anti-NMDAR encephalitis. Seronegativity is recognized in patients tested late or after immunotherapy initiation (Cellucci et al., 2020; Graus et al., 2016). The negative result also raises the differential of other AEs associated with ovarian teratoma, such as anti-AMPAR encephalitis. Anti-AMPAR encephalitis, however, is substantially rarer, typically presents at older ages, and shows a more neurological than psychiatric phenotype, with prominent seizures and disorientation rather than the manic-psychotic pattern observed here (Honnorat and Plazat, 2018). In our patient, moreover, the broad surface-antibody screening, which included AMPAR, was negative. The marked psychiatric and behavioral predominance of our patient's syndrome, including mania, hallucinations, and behavioral disinhibition, fits the anti-NMDAR profile. Diagnosis was therefore established through clinical features, EEG findings of bilateral dysfunction, and a partial but objective clinical and electrophysiological response to immunotherapy, consistent with pediatric AE criteria permitting probable diagnosis without antibody confirmation (Cellucci et al., 2020). Serial EEG improvement provided objective evidence of treatment response.

The cognitive and behavioral sequelae observed in our patient fit current pathophysiological models of anti-NMDAR encephalitis. Patient-derived antibodies cross-link and internalize synaptic NMDA receptors, impairing NMDA receptor-mediated transmission without complement deposition or substantial neuronal loss (Dalmau et al., 2019; Hughes et al., 2010). Autopsy studies of untreated cases show that the inflammatory infiltrate, dominated by plasma cells and CD3+/CD8-helper T cells, concentrates in the amygdala, hippocampus, and basal ganglia, with NMDAR loss correlating with disease severity (Zrzavy et al., 2021). This anatomical pattern mirrors the cardinal symptoms of behavioral disturbance, memory dysfunction, and movement abnormalities at presentation, all of which our patient exhibited.

After the inflammatory phase resolves, patients enter a recovery stage marked by residual deficits in executive function, attention, working memory, and sleep, often persisting for months to years (Dalmau et al., 2019). Structural and functional imaging studies have linked these deficits to hippocampal atrophy, altered frontoparietal connectivity, and superficial white matter damage in frontal and temporal lobes (Dalmau et al., 2019). Notably, conventional MRI is normal in a substantial proportion of patients despite measurable cognitive impairment (Heine et al., 2021), as in our patient whose 2024 MRI showed no structural abnormalities, which highlights the value of structured neuropsychological assessment over imaging alone for documenting residual disease burden. Our patient's profile of preserved verbal comprehension alongside disproportionate impairment in working memory, divided and alternating attention, processing speed, and verbal episodic memory aligns with this pattern of frontal-subcortical and hippocampal compromise. This multidomain profile is consistent with known long-term sequelae but is rarely documented with formal testing in individual case reports (Finke et al., 2012; Heine et al., 2021). A recent report in adult patients used adjunctive NMDAR antagonists for residual catatonic features (Kim et al., 2024), but pediatric cases with documented long-term neuropsychological profile and structured rehabilitation remain scarce. The substantial delay between symptom onset and definitive immunotherapy in our patient may have contributed to the severity of these residual deficits, and their persistence six years on, alongside social-communicative impairments meeting ASD criteria, may reflect both the duration of untreated inflammation and the developmental vulnerability of a brain still undergoing critical maturation.

The biological family history of psychiatric illness complicates causal attribution. Familial schizophrenia and other severe psychiatric disorders confer genetic vulnerability that could have contributed to preexisting behavioral difficulties or to the cognitive trajectory following acute illness. The abrupt subacute deterioration in 2018, the demonstrable response to immunotherapy, and the topographic match between symptoms and known inflammatory distribution nonetheless support an encephalitic contribution to current deficits.

The identification of ASD raises clinically relevant questions. Social-communicative difficulties and behavioral rigidity can appear both in anti-NMDAR encephalitis and ASD, and distinguishing the two is challenging without pre-illness developmental data (Dalmau et al., 2019). Longitudinal follow-up and serial neuropsychological evaluation - at approximately 24-month intervals, as recommended in the 2024 assessment - may help clarify this through differential recovery trajectories.

As the patient enters adolescence, the anticipated course is one of gradual but incomplete cognitive recovery, with persistent executive, attentional and academic difficulties alongside the social-communication profile. Reported pediatric cohorts describe residual attentional deficits and excessive fatigue years after anti-NMDAR encephalitis, with implications for educational attainment, independence and quality of life (De Bruijn et al., 2018; Titulaer et al., 2013). These expectations argue for structured transition planning, continued educational accommodation and periodic neuropsychological review.

The treatment course also highlights the need for coordinated, long-term multidisciplinary care. Acute immunotherapy stabilized the encephalitic process, but sustained improvement required careful psychiatric and neurological management with clozapine for psychosis, topiramate for seizures, and methylphenidate for attention. Combined with speech and language therapy, occupational therapy, educational accommodations, and psychological support, this integrated approach represents a reasonable model for pediatric AE (Dalmau et al., 2019; Titulaer et al., 2013). Evidence specifically supporting structured neuropsychological rehabilitation in AE remains limited, and current practice is largely extrapolated from acquired brain injury; the scarcity of pediatric rehabilitation data has been highlighted in a recent scoping review (Galioto et al., 2024).

The cognitive profile documented here aligns with the domains most consistently impaired across AE cohorts: verbal and visual episodic memory, attention and working memory, processing speed, and executive function, identified in a recent scoping review, which also noted the scarcity of pediatric data and the absence of studies in paraneoplastic presentations (Galioto et al., 2024). It is likewise consistent with prospective pediatric follow-up showing persistent attentional deficits and excessive fatigue years after anti-NMDAR encephalitis; notably, in that cohort treatment delay did not consistently predict neuropsychological outcome (De Bruijn et al., 2018), so the contribution of the protracted diagnostic delay in our patient is best regarded as a plausible but unproven factor. More broadly, the case can be read within an emerging immunopsychiatric framework (Miller et al., 2025; Sforzini and Pariante, 2025), in which earlier initiation of immunotherapy has been associated with better functional outcomes across transdiagnostic neuroimmune presentations, including antibody-negative cases (Scott et al., 2025).

From the patient's and caregiver's perspective, gathered during ongoing psychiatric follow-up, the patient regards herself as relatively well: she completed secondary education under an adapted curriculum, took the national university entrance examination with accommodations and, although unsuccessful, coped without clinical destabilization, redirecting her aspirations toward theater studies. She nonetheless rejects the diagnosis of ASD, which she experiences as distressing, and shows limited insight into her condition, while her adoptive mother reports intense apprehension about her daughter's future and, through her own psychotherapy, growing awareness of how mother-daughter dynamics influence the patient's irritability and mood.

Several limitations should be acknowledged together. Antibody testing was performed on a single occasion, in serum and CSF, more than 18 months after symptom onset and after corticosteroid initiation, which reduces sensitivity and precludes definitive serological confirmation. Brain MRI was not obtained during the acute illness. Pre-illness developmental data were limited by the adoption history which, together with a biological family history of severe psychiatric illness, constrains attribution of the neurodevelopmental findings to the encephalitis rather than to preexisting conditions. The autism diagnosis rested on a quantitative, school-rated instrument (SRS-2) integrated with clinical assessment rather than on gold-standard diagnostic instruments (ADOS-2, ADI-R), which were not available; no standardized adaptive-functioning measure was administered, so a co-occurring disorder of intellectual development could not be confirmed or excluded; and ADHD was not formally excluded by a dedicated protocol. Structured neuropsychological rehabilitation was recommended but its implementation could not be verified. Finally, the neuropsychological data derive from a single time point, so the trajectory of recovery could not be characterized.

## 4 Conclusions

This case demonstrates that seronegative pediatric anti-NMDAR encephalitis can leave a documented multidomain neuropsychological profile six years after symptom onset, even when conventional neuroimaging remains normal. The convergence of executive, attentional, working memory, and processing speed deficits with reported behavioral disturbance, memory dysfunction, and movement abnormalities at presentation maps onto the inflammatory distribution described in autopsy studies of untreated cases. In pediatric patients with incomplete developmental records, the differential between encephalitis sequelae and preexisting conditions may remain irresolvable, but this does not diminish the importance of formal neuropsychological assessment for guiding rehabilitation. For clinical practice, an abrupt neuropsychiatric change in a child – particularly a treatment-refractory or atypical psychiatric presentation accompanied by seizures, movement abnormalities or fluctuating cognition – should prompt early consideration of autoimmune encephalitis, antibody testing in both serum and CSF before immunotherapy where feasible, tumor screening (including pelvic imaging) in female patients, and formal neuropsychological assessment during follow-up even when MRI is normal. Seronegativity should not exclude the diagnosis, and structured cognitive assessment, rather than imaging, is often what reveals the residual burden and directs rehabilitation.

## CRediT authorship contribution statement

**Felipe Dalvi-Garcia:** Writing – review & editing, Writing – original draft, Methodology, Investigation, Data curation, Conceptualization, Funding acquisition. **Iolanda de Salles Fonseca Carvalho:** Writing – review & editing, Writing – original draft, Supervision, Methodology, Investigation, Data curation, Funding acquisition, Resources. **Julia Valeriano de Almeida:** Writing – review & editing, Investigation, Data curation, Funding acquisition, Resources. **Roozemeria Pereira Costa:** Writing – review & editing, Investigation, Formal analysis, Funding acquisition. **Ligia Caldeira da Silva:** Writing – review & editing, Investigation, Funding acquisition. **Julia Nunes Perez Fandiño:** Writing – review & editing, Supervision, Funding acquisition.

## Ethics declaration

Written informed consent to take part in the study and to publish the article has been obtained from all participants or their legal representatives. The privacy rights of participants have been observed.

This study was performed in compliance with relevant laws, regulatory frameworks and guidelines where the research took place. This study was approved by the Research Ethics Committee of Gaffrée e Guinle University Hospital. (Approval No. 77625623.0.0000.5258, February 29, 2024)

This research follows the CARE guidelines and the CARE checklist.

## Previous presentation

A preliminary version of this case was presented as a poster abstract at the World Congress of the International Association for Child and Adolescent Psychiatry and Allied Professions (IACAPAP), Rio de Janeiro, Brazil, May 20-24, 2024.

## Declaration of generative ai and ai-assisted technologies in the manuscript preparation process

During the preparation of this work the authors used Claude (Anthropic) in order to improve the language and readability of the manuscript and to assist with organizing the content. After using this tool, the authors reviewed and edited the content as needed and take full responsibility for the content of the published article.

## Funding and acknowledgments

This work received no specific funding from any agency in the public, commercial, or not-for-profit sectors.

## Declaration of competing interest

The authors declare that they have no known competing financial interests or personal relationships that could have appeared to influence the work reported in this paper.

## Data Availability

The data that has been used is confidential.
